# Book Reviews

**Published:** 1898-06

**Authors:** 


### BOOK REVIEWS.

---

Prompt Aid to the Injured. Designed for Military and Civil Use. By Alvah H. Doty, M.D., Late Attending Surgeon to Bellevue Hospital Dispensary, Late Major and Surgeon Ninth Regiment, New York. Second Edition, Revised and Enlarged. Price $1.50. D. Appleton & Co., New York. [Southern Agency, Atlanta.]

"The object of this manual is to instruct those who are desirous of knowing what course to pursue in emergencies, in order that sick or injured may be temporarily relieved. Special effort has been made to so arrange the matter and introduce such points as will be of use to the ambulance corps connected with military organizations."

The first seven chapters are devoted to the essentials of anatomy and physiology. Then come chapters on bandages and dressings, antiseptics, disinfectants and deodorants. The next chapters contain practical suggestions upon rendering prompt and intelligent aid in cases of contusions and wounds, hemorrhage, fractures, sprains and dislocations, burns, scalds and frost-bite, shock and syncope, concussion of the brain, apoplexy, intoxication, epilepsy, asphyxia and drowning, poisoning, etc. A chapter is devoted to the hygiene of baths, clothing, food, water, air and exercise. The last chapter describes the proper methods of transporting the wounded, and includes the "Drill Regulations for the Hospital Corps, U. S. Army." The latter is particularly useful just at this time.

---

Brief Essays on Orthopedic Surgery. By Newton M. Shaffer, M.D., Surgeon-in-Chief to the New York Orthopedic Dispensary and Hospital, etc. D. Appleton & Co., Publishers, 72 Fifth ave., New York.

This little book of eighty-one pages is a collective republication of a number of essays which this author has written for various medical journals in the last fourteen years. These separate titles are: "The Present Status of Orthopedic Surgery;" "What is Orthopedic Surgery?"; "The Definition and Scope of Orthopedic Surgery;" "The Relation of Orthopedic Surgery to General Surgery;" "The Present Needs and Future Demands of Orthopedic Surgery;" "The Operative Side of Orthopedic Surgery;" "Is Orthopedic Surgery to become an Obsolete Specialty, with Remarks on Specialism." Those interested in orthopedic literature will find these essays quite interesting and instructive.

---

COLLIER'S WEEKLY, MAY 28TH.

Sixty-nine pictures appear in the current number of *Collier's Weekly;* eight of them are portraits of Gladstone at different periods of his life, and four have other subjects, but more than fifty are objects, scenes and individuals made specially prominent and interesting by the war with Spain. More than a month ago Mr. Hare, of the *Weekly's* photographic staff, went, with two other journalists, to the camp of the insurgent general Gomez with the first news of American intervention in Cuba. Mr. Hare wrote a description of the trip and took scores of photographs; the first instalment of his narrative, with about twenty of his pictures, appears in the current number; a double-page picture of Gomez, in his hammock, chatting with his American visitors, is from a painting by Gilbert Gaul, after one of Mr. Hare's photographs. For frontispiece the paper displays the conning-tower of the torpedo boat "Winslow," showing the results of Spanish shots at Cardenas. This battle itself is the subject of a full-page picture, after a painting by Ritschel; the funeral of the "Winslow's" dead, and portraits of the survivors, are the subjects of several pictures. There are camp scenes from New Orleans, Chattanooga and Tampa, as well as from some State camps. A good map of the scene of naval maneuvers, fine portraits of Admiral Cervera of the Spanish fleet, Commodore Watson (who fought with Farragut on the "Hartford"), Captains Barker, Philip, Lamberton and Dickens of the navy. Ensign Bagley, who was killed on the "Winslow," Cadet Pettengill, who fired the first shot of the war, Col. Fred Grant, Governor Black of New York, and pictures of several vessels that have achieved distinction in one way or other, add to the value of the number.

